# 

**Published:** 1889-07

**Authors:** 


VOL. X. JULY, 1889. No. 7.

### THE
### International Dental Journal

A MONTHLY PERIODICAL

DEVOTED TO

### Dental and Oral Science.

W. XAVIER SUDDUTH, M.D., D.D.S.,
EDITOR.
Office, 716 Filbert Street, Philadelphia.

PUBLISHED BY THE
INTERNATIONAL DENTAL PUBLICATION COMPANY,
51 WEST THIRTY-SEVENTH STREET, NEW YORK CITY,
—AND—
716 FILBERT STREET, PHILADELPHIA.

ENTERED AT PHILADELPHIA POST-OFFICE AS SECOND-CLASS READING MATTER.

$2.50 per Annum, in Advance. Single Copies, 25 Cents.

Printed by J. B. Lippincott Company, Philadelphia.

